# Potentially traumatic experiences pre-migration and adverse pregnancy and childbirth outcomes among women of Somali- and Kurdish-origin in Finland

**DOI:** 10.1186/s12884-023-05906-w

**Published:** 2023-08-17

**Authors:** Satu Majlander, Tarja I. Kinnunen, Eero Lilja, Mika Gissler, Anu E Castaneda, Lara Lehtoranta, Päivikki Koponen

**Affiliations:** 1 Department of Public Health and Welfare, Unit of Equality, Finnish Institute for Health and Welfare, Mannerheimintie 166, PL 30, Helsinki, 00271 Finland; 2 Unit of Health Sciences, Faculty of Social Sciences, Tampere University, Arvo Ylpön katu 34, Tampere, 33014 Finland; 3 Unit of Health Sciences, Faculty of Social Sciences, Tampere University, Arvo Ylpön katu 34, Tampere, 33014 Finland; 4 Department of Knowledge Brokers, Finnish Institute for Health and Welfare, Mannerheimintie 166, PL 30, Helsinki, Helsinki, 00271 Finland; 5 Region Stockholm, Academic Primary Health Care Centre, Stockholm, Sweden; 6 Department of Molecular Medicine and Surgery, Karolinska Institutet, Stockholm, Sweden; 7 Research Centre for Child Psychiatry and Invest Research Flagship, University of Turku, Turku, Finland; 8 Department of Public Health and Welfare, Population Health Unit, Finnish Institute for Health and Welfare, Mannerheimintie 166, PL 30, Helsinki, 00271 Finland; 9 Department of Public Health and Welfare, Finnish Institute for Health and Welfare, Mannerheimintie 166, PL 30, Helsinki, 00271 Finland

**Keywords:** Reproductive health, Migrant, Women, Potentially traumatic experiences, Female genital mutilation/cutting (FGM/C)

## Abstract

**Background:**

Women in precarious conditions in their countries of origin, especially those who have left the country as refugees, may have been victims of serious mental and physical violence. These potentially traumatic experiences may threaten women’s reproductive health. This study examines the prevalence of potentially traumatic experiences pre-migration and female genital mutilation/cutting (FGM/C) and their associations with adverse reproductive outcomes among migrant women of Somali- and Kurdish-origin who have been pregnant in Finland.

**Methods:**

Survey and register data of the participants of the Finnish Migrant Health and Wellbeing Study (Maamu), conducted in 2010–2012, were used. Women of 18 to 64 years of age, 185 Somali- and 230 Kurdish-origin, who had at least one pregnancy or birth in Finland were included in the analysis. The survey data were linked to the Finnish Medical Birth Register, the Register of Induced Abortions, and the Care Register for Health Care until 2018. For each outcome, logistic regression was used and adjusted for age, body mass index, time lived in Finland, and the number of births.

**Results:**

A total of 67% of Somali-origin and 71% of Kurdish-origin women had experienced potentially traumatic experiences pre-migration and 64% of Somali- and 32% of Kurdish-origin women had also undergone FGM/C. In Kurdish-origin women, complications during pregnancy (e.g. bleeding in the first trimester, known or suspected fetal abnormality, signs of fetal hypoxia, death of the fetus and other problems) were significantly more common among women without potentially traumatic experiences (70%) than among women with potentially traumatic experiences (48%) (p-value 0.005). No associations between potentially traumatic experiences or FGM/C and other adverse reproductive outcomes were observed among Somali- or Kurdish-origin women.

**Conclusion:**

Past trauma is common among Somali- and Kurdish-origin women and this needs to be evaluated in maternity care. However, we found no association between potentially traumatic experiences pre-migration and adverse reproductive outcomes.

**Supplementary Information:**

The online version contains supplementary material available at 10.1186/s12884-023-05906-w.

## Background

Women of migrant origin, especially those with a refugee background, have an increased risk of adverse reproductive outcomes (e.g., preterm birth, caesarean section, low birthweight, and elevated perinatal mortality) [1–4]. Register-based studies show that women of Sub-Saharan African, South Asian, and East Asian origin are at a greater risk of emergency caesarean delivery, preterm birth, low birthweight and lower 5-minute Apgar scores for newborns, and Somali-origin women have an increased risk for any birth complication compared to Finnish-origin women [5, 6]. A population-based study linking the Medical Birth Registry of Norway to Statistics Norway included the first registered birth during the study period (2006–2010) of women from Somalia, Iraq, Afghanistan, and Kosovo and ethnic Norwegians. Women from Somalia were most at risk for adverse obstetric outcomes. Compared with ethnic Norwegians, they had increased odds ratios (OR) for emergency cesarean section, post term birth, meconium-stained liquor, and having a small-for-gestational-age infant [7]. A retrospective study by Gibson-Helm et al. (2015) studied maternal health, antenatal care, and pregnancy outcomes among refugee women (born in Asian humanitarian source countries) and non-refugee women born in Asian. The results showed that women born in Afghanistan, Bhutan, Iraq, and Myanmar had poorer maternal health. In addition, women from South Asian humanitarian source countries were less likely to participate in antenatal care and were at higher risk of prolonged pregnancy [8].

A previous register-based study in Finland by Väisänen et al. (2022) studied interactions between migrant origin and individual socioeconomic status and the time lived in the host country in relation to reproductive health. The results of the study showed that the impact of time after immigration varied, and its effect depended on country of birth and the outcome studied. Migrants from low- and lower-middle-income countries had a higher risk of preterm birth than Finnish-born women [9].

Potentially traumatic experiences such as conflicts and war, are usually unexpected and unpredictable events in women’s lives. Women have an increased risk of experiencing violence during conflicts [10]. A Finnish study found a high prevalence of potentially traumatic experiences in the former home country among Somali and Kurdish origin women (69% and 72%) [11]. Common consequences of violence on the sexual and reproductive health of women include unintended/unwanted pregnancies, abortions/unsafe abortions, pregnancy complications/miscarriages and sexual dysfunction [12].Potentially traumatic experiences, such as being tortured, raped, persecuted, or experiencing personal losses, can have severe long-term consequences on women’s health [13], both immediately and long term [12, 14]. These include injuries, mental health conditions such as anxiety and depression [15, 16], reproductive health problems including adverse outcomes in pregnancy [17] and sexually transmitted diseases [18]. Furthermore, the effects of traumatic experiences are associated with chronic diseases, such as high blood pressure and increased body mass index [19].

Context-specific factors such as poverty and experiences of inequality, may influence the severity of the effects of violence [12]. A review article that compiled existing research data shows that traumatized women have a higher susceptibility to post-traumatic stress disorder symptoms associated with potentially traumatic experiences such as miscarriage or physical and mental changes during pregnancy [20]. Previous studies in Finland have reported that pregnancies of women who suffered from major depression during pregnancy more frequently resulted in adverse perinatal outcomes, such as stillbirth, preterm birth, fetal macrosomia or being small for gestational age, low Apgar scores, fetal hypoxemia or hypoxia, neonatal need for intensive care and major congenital anomalies, than women without major depression [21, 22].

One potentially traumatic experience in a woman’s life is female genital mutilation/cutting (FGM/C). It is performed within a wide range of African ethnic groups, in Asia and in some Arab countries. It is estimated that at least three million girls are at risk of undergoing the practice every year [23–25]. According to WHO, more than 200 million girls and women alive today have undergone FGM/C in 30 countries in Africa, the Middle East and Asia, where FGM/C is practiced [26]. In Somalia, 99% of girls and women have undergone FGM/C, and most women (64%) have been subjected to FGM/C Type III (excision of part or all of the external genitalia and stitching or narrowing of the vaginal opening) [27]. FGM/C is widely practiced in Iraqi Kurdistan region, which is inhabited mostly by Muslim Kurds. The prevalence of FGM/C in Iraqi Kurdistan region is around 40% and almost all (99.6%) women who have undergone FGM/C, have been subjected to Type I FGM/C (partial or total removal of the clitoris and/or the prepuce) [28].

FGM/C is a harmful practice and influences women`s reproductive health, increasing the risk of obstetric hemorrhage, prolonged labor and overall difficult delivery, and in addition recurrent urinary tract infections and infertility [24, 29, 30]. Being mutilated can cause psychological impacts during pregnancy and might raise concerns on the ability of health professionals to deal with FGM/C during pregnancy and labor [25]. The psychological impact of FGM/C can have lasting life-long consequences [25]. FGM/C is an emerging issue in several countries, including Finland. In 2012 the prevalence of FGM/C was 69% among women of Somali-origin and 32% among women of Kurdish-origin living in Finland [31]. The effects of potentially traumatic experiences among migrant-origin women living in Finland are not well known.

This study examines the prevalence of potentially traumatic experiences pre-migration and FGM/C among migrant women of Somali- and Kurdish-origin who have been pregnant in Finland, and whether they are associated with adverse reproductive outcomes (such as miscarriages, induced abortions, preterm births and severe complications during pregnancy and childbirth) in at least one pregnancy or birth in Finland.

## Methods

### Study population and Maamu survey

The sample of Somali- and Kurdish-origin migrant women living in Finland are from the Finnish Migrant Health and Wellbeing Study (Maamu). It is a comprehensive cross-sectional interview and health examination survey conducted by the Finnish Institute of Health and Welfare (THL) in 2010–2012, with linked register data. A more detailed description of survey methods has been provided elsewhere [32, 33]. Briefly, a stratified random sample of 1000 Somali-, 1000 Kurdish- and 1000 Russian-origin adults was selected from the National Central Population Registry, aged 18 to 64 years, and living in six Finnish cities (Helsinki, Espoo, Vantaa, Turku, Tampere, and Vaasa).

**Fig. 1:**
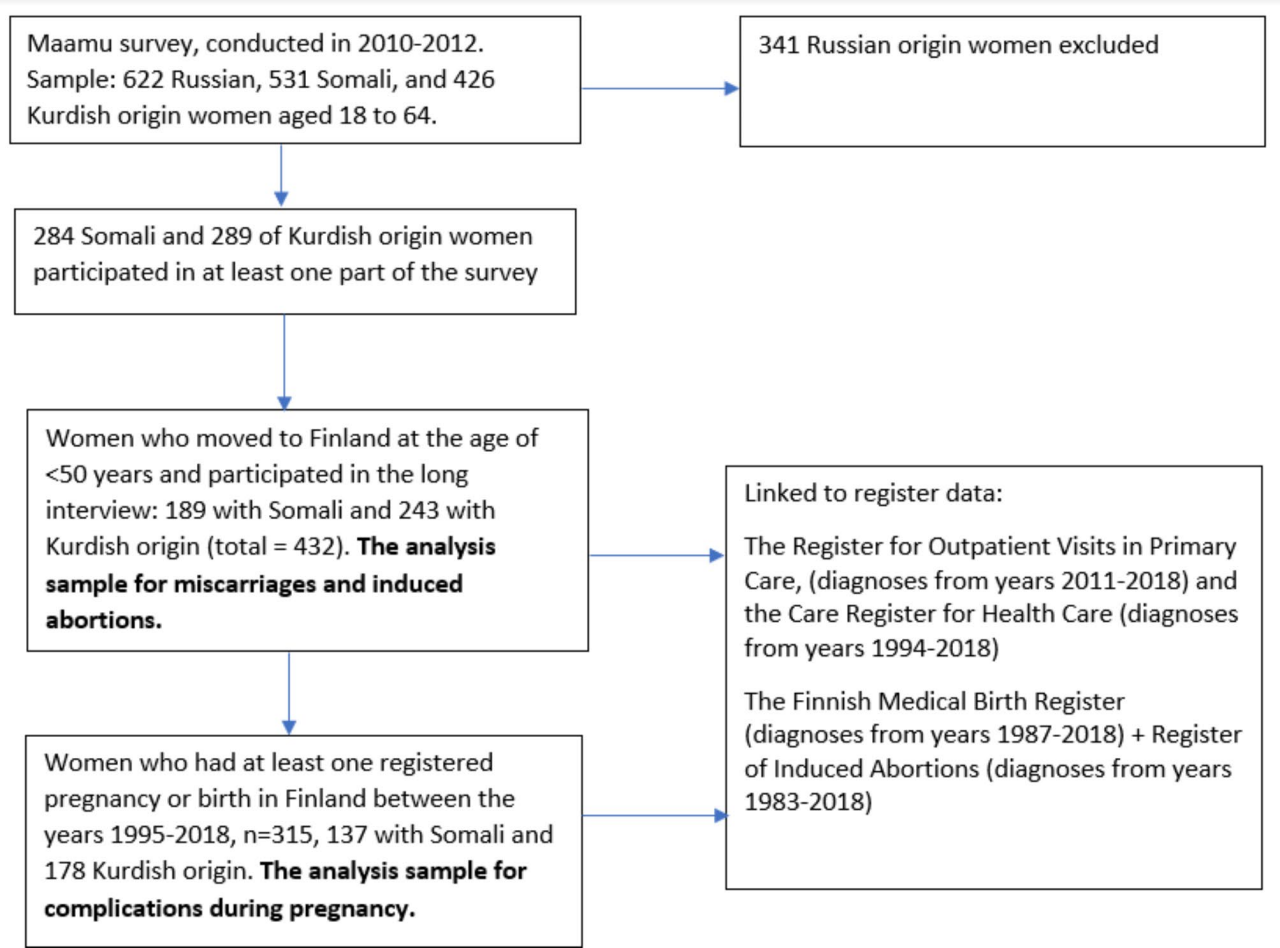
Flow chart

For this study, the selected subjects were Somali- and Kurdish-origin women who had arrived in Finland at the age of < 50 years and participated in the long interview of the Maamu study (Fig. 1). Russian women were excluded from this study as the questions on FGM/C were not addressed to them and the proportion of those who had experienced trauma was low (20%). To study potentially traumatic experiences, miscarriages and induced abortions, the sample was narrowed down to women with at least one self-reported and/or registered pregnancy or birth in Finland in 1995–2018 (Fig. 1), which resulted in 189 Somali- and 243 Kurdish-origin women to be included in this study. To study complications during pregnancy and birth the sample was narrowed down to women who had at least one registered pregnancy or birth in Finland between the years 1995–2018 (137 Somali- and 178 Kurdish-origin) as all information on complications came from registers. All pregnancies and births of these women in Finland were included in the analysis, as the coverage of birth and hospital register data is good in Finland.In the Maamu survey, interviews were conducted face-to-face in the native language of the participants (Somali or Kurdish language). The Maamu survey with the register linkages were approved by the coordinating Ethical Committee of Helsinki and Uusimaa Hospital District (decision 325/13/03/00/2009) and by the organizations keeping the register data. All participants gave written informed consent.

### Register data

Data were obtained from four registers from the years 1995–2018: 1) the Finnish Medical Birth Register (MBR), 2) the Register of Induced Abortions, and 3) the Register for Outpatient Visits in Primary Care and 4) the Care Register for Health Care. The survey data were linked to the register data for all participants using the national registration number assigned to all residents in Finland. More detailed information on this combined data has been described elsewhere [32]. Linkage to the registers allowed analyses on e.g., birth years, birth outcomes, diagnoses, and hospital visits not asked for in the survey and occurring after the survey.

1. The MBR contains data on all live births and stillbirths (after 22 gestational weeks or with the baby’s birthweight at least 500 g) that have occurred in Finland as well as information on the number of previous pregnancies, deliveries and induced abortions that have been self-reported in maternity health services.
2. The Register of Induced Abortions contains information on all legally induced abortions in Finland. The register also includes information on the number of previous abortions and previous pregnancies that have been self-reported when having the abortion. The physician performing an abortion is required to report the case to the THL.
3. The Register for Outpatient Visits in Primary Care includes basic information on all visits to public primary health care.
4. The Care Register for Health Care contains data on patients discharged from inpatient care and day surgeries in all hospitals, as well as specialized outpatient care in all public hospitals in Finland.

### Definition of variables

In the Maamu survey potentially traumatic experiences in the country of origin were addressed with eight questions about different conflict situations (adapted from Harvard Trauma Questionnaire (HTQ, 18)) [11]: *“Have you experienced the following unpleasant events in your former home country: 1) Experienced a combat situation in a war? 2) Been the victim of a natural disaster such as an earthquake, a flood, or a fire? 3) Witnessed violent death or serious injury? 4) Experienced sexual violence? 5) Been a target of a serious physical attack or harm? 6) Been detained or kidnapped? 7) Been tortured? 8) Experienced some other form of horrible violence?”.* Response items were *“yes”* and *“no”*. A binary composite variable was calculated by categorizing the “yes” responses to any of these questions into ‘a potentially traumatic experience‘ and “no” responses to all eight items to ‘no potentially traumatic experiences’. The composite variable was used to examine the association between trauma and reproductive complications. Reproductive health issues were asked mainly by a female interviewer or nurse. The FGM/C status of women was asked among other reproductive health issues in the interview with the options *“yes”* and *“no”.* This was complemented by registered diagnoses suggestive of FGM/C. Information on the type of FGM/C could not be obtained from the data.

All self-reported and/or registered births, induced abortions, or miscarriages after moving to Finland were studied. An outcome in at least one pregnancy and birth was included in the analyses of this study. In the Maamu survey the numbers of births were asked: *“How many births have you had? Include all births, including Caesarean sections.”* The numbers of induced abortions were asked: *“Have you had any induced abortions and how many?”*, and miscarriages were asked: *“How many miscarriages have you had?”* In this study, the register information complements the survey information for those variables for which data were available from both sources. The outcome variables from the survey and registers and potential confounding factors are defined in Tables 1 and 2. We used several ICD-10 diagnosis codes (International Statistical Classification of Diseases and Related Health Problems) and ICPC2 codes (International Classification of Primary Care) classified for events during pregnancy and childbirth. (Appendix, Table 1). Mental health diagnoses were also checked from the register, but the number of women with diagnosed mental health disorders was too low for analysis. When examining the association between trauma/FGM/C and complications, we combined serious complications during pregnancy into one variable (complications at any pregnancy event) and childbirth into another (complications at any birth event).

### Statistical analysis

Inverse probability weights were used in all analyses to reduce the effect of non-response bias and to account for different sampling probabilities [34]. The weights were calculated using registered information from the National Central Population Registry on age, sex, marital status, migrant group, and municipality [33]. Variance estimation was based on Taylor linearization method throughout this study, accounting for the stratified sampling. SAS 9.4 software was used for constructing outcome variables and calculating crude values, whereas SUDAAN 11.0.3 was used for data analysis.

We present the weighted distributions, their logit-transformed 95% confidence intervals and the crude number of events for the variables. The association between potentially traumatic experience pre-migration, FGM/C and the outcome variables were first analyzed with univariate logistic regression (Model 1). In Model 2, the associations for potentially traumatic experience pre-migration were adjusted for age at the latest pregnancy/birth (≥ 40 vs. <40 years), body mass index (≥ 25 vs. < 25 kg/m^2^), time lived in Finland (≤ 6 vs. >6 years), hypertension during any pregnancy (yes vs. no), gestational diabetes in any pregnancy (yes vs. no), and the number of births (0, 1–2, ≥ 3). The association between FGM/C and the outcome variables were adjusted for age at the latest pregnancy/birth, time lived in Finland and number of births.

The differences between the groups were reported using odds ratios (OR) and their 95% confidence intervals as well as *p*-values based on Satterthwaite-adjusted F-Statistic. The model-adjusted distributions were calculated using predicted marginals [35].

## Results

Descriptive information of the women studied at the time of the survey data collection is presented in Table 1. Most women in both groups were married, not employed, and had lived in Finland longer than five years. Nearly a third were overweight (measured in the survey). Among Somali-origin women, 28% had gestational diabetes (GDM) and 14% hypertension during any pregnancy in Finland. Among Kurdish-origin women 22% had GDM and 5% had hypertension. The average number of outpatient and inpatient visits during the latest pregnancy in Finland was 13 among Somali-origin women and 16 among Kurdish-origin women.

In total, 67% of Somali- and 71% of Kurdish-origin women had experienced a potentially traumatic experience pre-migration (Table 2). As many as 64% of Somali-origin women and 32% of Kurdish-origin women had undergone FGM/C. A majority (73%) of women with a Somali-origin and almost half (48%) of Kurdish-origin women had three or more births. In total 8% of Somali-origin women and 19% of Kurdish-origin women had experienced two or more induced abortions. In women with at least one registered pregnancy or birth in Finland, 69% of Somali-origin and 56% of Kurdish-origin women had complications during pregnancy (including bleeding in the first trimester and complications related to fetus) and 61% of Somali-origin women and 50% of Kurdish-origin woman had complications during childbirth (including preterm birth, failed induction of labor, labor and delivery complicated by intrapartum hemorrhage and fear of childbirth) (Table 2.). A quarter (26%) of Somali- and 31% of Kurdish-origin women had an assisted birth, and 27% of Somali- and 17% Kurdish-origin women had tears, hemorrhage, and other complications during childbirth.

No significant differences were seen within or between those with or without potentially traumatic experiences in Model 1 (Table 3). An equal proportion (68%) of Somali-origin women with potentially traumatic experiences and without potentially traumatic experiences had complications during pregnancy. On the other hand, 48% of Kurdish-origin women with potentially traumatic experiences and 65% of women without potentially traumatic experiences had complications during pregnancy (p = 0.023). Very few statistically significant differences were observed between women with potentially traumatic experiences and women without potentially traumatic experiences, also when adjusting for confounders in Model 2 (Table 3). Among Kurdish-origin women, complications during pregnancy were more common in women without potentially traumatic experiences than in women with potentially traumatic experiences (OR 0.34, Cl 0.16–0.72).

**Table 1:** Participants’ characteristics by the country of origin

	Somali		Kurdish	
	*n = 185	% (95% Cl)	*n = 230	% (95% Cl)
	**n = 131		**n = 158	
**Age** *				
18–29 years	50	21.9 (16.5–28.6)	42	17.2 (13.2–22.0)
30–39 years	71	37.7 (30.6–45.3)	129	54.6 (48.3–60.3)
40–53 years	66	40.4 (33.2–48.1)	71	28.2 (23.3–33.7)
**Marital status** *				
Unmarried/divorced/widow	57	32.7 (25.7–40.4)	71	32.8 (27.5–38.7)
Married/civil union	128	67.3 (59.6–74.3)	172	67.2 (61.3–72.5)
**Education** *				
Lower than upper secondary school	160	82.6 (75.6–87.9)	139	56.4 (50.6–62.0)
Upper secondary school	26	17.4 (12.1–24.4)	103	43.6 (38.0–49.3)
**Employment status** *				
Working	25	14.9 (10.5–21.6)	75	30.5 (25.4–36.1)
Others	159	85.1 (78.5–89.7)	166	69.5 (63.9–74.6)
**Age when moved to Finland** #				
> 18 years	122	64.2 (56.4–71.4)	180	76.8 (71.1–81.6)
≤ 18 years	63	35.8 (28.6–43.6)	50	23.3 (18.4–28.9)
**Time lived in Finland** #				
≤ 5 years	50	22.3 (16.8–29.1)	42	17.8 (13.6–22.9)
6–14 years	71	37.4 (30.3–45.1)	129	55.1 (49.1–61.0)
≥ 15 years	66	40.1 (33.0–48.0)	71	27.1 (22.2–32.7)
**Maternal health service use and health**				
The number of outpatient and inpatient visits during the latest pregnancy in Finland, mean **	131	12.6	158	16.2
Body mass index BMI, mean #	171	27.8	229	26.8
Gestational diabetes mellitus diagnosed in Finland**	37	27.5 (20.4–36.1)	39	22.3 (17.1–28.5)
Hypertension during pregnancy in Finland**	18	14.0 (8.9–21.3)	8	4.7 (2.4–8.3)

Numbers, prevalence (%), and 95% confidence intervals (CI), or means

# Survey data (Somali n = 284, Kurdish n = 289)

* Combined survey and register data from Maamu study, MBR and Register of Induced Abortions and The Care Register for Health Care

**Data from MBR, Register of Induced Abortions and The Care Register for Health Care

**Table 2:** Female genital mutilation/cutting (FGM/C), trauma, and complications during pregnancy and birth

	Somali		Kurdish	
	*n = 185	% (95% Cl)	*n = 230	% (95% Cl)
	**n = 131		**n = 158	
**Potentially traumatic experience** #				
self-reported potentially traumatic experience	109	66.7 (59.2–73.5)	174	71.4 (65.9–76.4)
**Female genital mutilation/cutting (FGM/C)** *				
undergone FGM/C	119	64.2 (56.6–71.1)	77	32.4 (27.2–38.1)
**Reproductive health during lifetime**				
**Births** *				
no births	27	13.0 (8.6–19.1)	27	12.1 (8.7–16.7)
1–2 birth	25	13.3 (8.9–19.4)	92	39.8 (34.3–45.6)
3 births or more	137	73.7 (66.5–79.9)	124	48.1 (42.4–53.8)
Miscarriages*				
none	102	55.8 (48.1–63.1)	156	65.2 (59.5–70.5)
1–2 miscarriages	40	20.6 (15.2–27.4)	42	17.2 (13.2–22.0)
3 or more	47	23.6 (17.8–30.7)	45	17.6 (13.7–22.3)
**Abortions** *				
none	163	85.2 (78.7–90.1)	160	66.4 (60.7–71.6)
one	14	6.9 (3.9–12.0)	38	15.1 (11.4–19.7)
2 or more	12	7.9 (4.4–13.6)	45	18.6 (14.5–23.5)
**Complications** (diagnosed in Finland after migration)†				
At least one complication during pregnancy **, †	121	68.8 (61.5–75.2)	134	55.5 (49.7–61.2)
At least one complication during childbirth **, †	108	61.1 (53.5–68.2)	120	49.9 (44.1–55.7)
Complications during the first trimester of pregnancy, bleeding **	30	20.9 (14.5–29.0)	29	16.5 (12.1–22.3)
Complications during pregnancy related to fetus**	56	42.2 (33.9–51.1)	49	28.2 (22.5–34.7)
Other complications during pregnancy **	50	36.2 (28.2–45.0)	52	30.5 (4.6–11.5)
Complications during pregnancy and birth**	78	61.9 (53.2–69.9)	63	35.7 (29.6–42.4)
Complications during birth, tears, haemorrage and other**	37	27.3 (20.1–35.9)	30	16.6 (12.1–22.3)
Complications during birth, assisted birth and other**	37	26.2 (19.2–34.6)	54	31.4 (25.4–38.1)

Numbers, prevalence (%), and 95% confidence intervals (CI)

# Survey data

* Combined survey and register data from Maamu study, MBR and Register of Induced Abortions and The Care Register for Health Care

**Data from MBR, Register of Induced Abortions and The Care Register for Health Care

† Detailed list of identified and included complications, see Appendix 1, Table 1

**Table 3:** Complications by trauma status and country of origin

	Logistic regression Model 1. Unadjusted				Logistic regression Model 2. Adjusted for mothers age (≥ 40), BMI (≥ 25), time lived in Finland (≥ 5) and number of births (≥ 3)					
	Somali		Kurdish		Somali			Kurdish		
	% (95% Cl)	p-value	% (95% Cl)	p-value	% (95% Cl)	p-value	OR	% (95% Cl)	p-value	OR
**Miscarriages, at least one during lifetime** *										
no trauma	48.9 (36.0–61.9)	0.384	33.8 (23.6–45.7)	0.810	55.7 (40.4–70.0)	0.342	1	45.7 (33.7–58.2)	0.406	1
trauma	41.8 (33.0–51.2)		35.3 (29.4–41.8)		46.4 (35.6–57.5)		0.65 (0.27–1.57)	40.2 (32.9–47.8)		0.74 (0.35–1.58)
**Induced abortions, at least one during lifetime** *										
no trauma	20.9 (11.7–34.7)	0.159	31.6 (22.3–42.5)	0.694	26.3 (14.1–43.6)	0.136	1	41.7 (29.0–55.6)	0.824	1
trauma	12.1 (7.1–19.9)		33.9 (27.9–40.5)		14.0 (7.9–23.4)		0.43 (0.14–1.30)	43.5 (35.9–51.4)		1.08 (0.53–2.21)
**Complications during pregnancy, at least one after migration** **										
no trauma	68.0 (52.8–80.1)	0.982	65.1 (53.2–75.4)	**0.023**	64.7 (48.9–77.9)	0.752	1	70.2 (57.8–80.3)	**0.005**	1
trauma	68.2 (56.8–77.8)		48.2 (40.3–56.3)		67.8 (56.2–77.5)		1.15 (0.47–2.78)	48.3 (40.3–56.4)		0.34 (0.16–0.72)
**Complications during birth, at least one after migration** **										
no trauma	69.1 (53.5–81.4)	0.613	68.4 (55.9–78.6)	0.096	72.0 (56.7–83.5)	0.859	1	69.3 (55.5–80.3)	0.116	1
trauma	73.6 (62.7–82.3)		55.7 (47.5–63.6)		73.6 (62.1–82.6)		1.09 (0.41–2.89)	56.5 (48.42–64.4)		0.55 (0.26–1.15)

Prevalence (%) and 95% confidence intervals (CI) and odds ratio (OR) (95% CI)

* Combined survey and register data from Maamu study, MBR and Register of Induced Abortions and The Care Register for Health Care

**Data from MBR, Register of Induced Abortions and The Care Register for Health Care

Among Somali-origin women, 46% of women with FGM/C and 41% of women without FGM/C had experienced at least one miscarriage and among Kurdish-origin women 37% of women with FGM/C and 34% of women without FGM/C had experienced at least one miscarriage, but the differences were nonsignificant (Table 4).

**Table 4:** Complications by female genital mutilation /cutting (FGM/C) status and country of origin

	Logistic regression Model 1. Unadjusted				Logistic regression Model 2. Adjusted for mothers age (≥ 40), BMI (≥ 25), time lived in Finland (≥ 5) and number of births (≥ 3)					
	Somali		Kurdish		Somali			Kurdish		
	% (95%Cl)	p-value	% (95%Cl)	p-value	% (95%Cl)	p-value	OR	% (95%Cl)	p-value	OR
**Miscarriages, at least one during lifetime** *										
no FGM/C	40.6 (28.5–53.9)	0.492	33.6 (27.4–40.4)	0.542	48.2 (33.5–63.2)	0.676	1	39.4 (31.9–47.5)	0.347	1
FGM/C	46.1 (37.1–55.3)		37.1 (28.0–47.3)		52.1 (41.6–62.4)		1.18 (0.52–2.67)	45.7 (34.8–56.9)		1.31 (0.69–2.47)
**Induced abortions, at least one during lifetime** *										
no FGM/C	20.9 (11.7–34.7)	0.159	34.3 (28.0–41.2)	0.739	20.4 (10.6–35.7)	0.739	1	42.4 (34.5–50.7)	0.988	1
FGM/C	12.1 (7.1–19.9)		32.4 (24.2–41.9)		17.9 (10.9–28.0)		0.84 (0.31–2.26)	42.5 (32.1–53.6)		1.00 (0.54–1.84)
**Complications during pregnancy, at least one after migration** **										
no FGM/C	69.5 (54.8–81.0)	0.960	57.5 (49.3–65.3)	0.980	69.3 (54.6–80.9)	0.980	1	57.7 (49.5–65.5)	0.198	1
FGM/C	69.1 (58.2–78.1)		46.4 (35.3–57.9)		69.1 (58.3–78.2)		0.98 (0.43–2.25)	48.7 (37.3–60.1)		0.67 (0.36–1.23)
**Complications during birth, at least one after migration** **										
no FGM/C	70.1 (55.0–81.8)	0.718	63.1 (54.9–70.7)	0.567	69.9 (54.5–81.8)	0.567	1	63.8 (55.6–71.2)	0.191	1
FGM/C	73.2 (62.5–81.7)		54.3 (41.8–64.7)		74.6 (64.4–82.7)		1.29 (0.53–3.11)	54.8 (43.2–65.9)		0.66 (0.36–1.22)

﻿prevalence (%) and 95% confidence intervals (CI) and odds ratio (OR) (95% CI)

* Combined survey and register data from Maamu study, MBR and Register of Induced Abortions and The Care Register for Health Care

**Data from MBR, Register of Induced Abortions and The Care Register for Health Care

Among Somali-origin women 12% of women with FGM/C and 21% of women without FGM/C had experienced at least one induced abortion. Among Somali-origin women with FGM/C and without FGM/C almost equal proportion (69–70%) had complications during pregnancy. No significant differences were seen in pregnancy or birth complications between either Somali- or Kurdish-origin women with or without FGM/C. As was the case with self-reported trauma, even when adjusting for confounders no statistically significant differences were observed between women with FGM/C and women without FGM/C in Model 2 (Table 4).

## Discussion

This study shows that many Somali- and Kurdish-origin women have underlying potentially traumatic experiences that need to be considered during pregnancy and in the treatment of childbirth. However, no significant differences were observed in the outcomes between women with potentially traumatic experiences and women without potentially traumatic experiences, apart from Kurdish-origin women without potentially traumatic experiences having a higher rate of complications during pregnancy when adjusted for confounders. This was a contrary finding from previous studies, where migrant women with potentially traumatic experiences have an increased risk of many adverse reproductive outcomes [12, 16, 20]

For example Lev-Wiesel et al. [36] have shown that traumatized women have a higher probability of experiencing complications during childbirth, including cesarean section, preterm labor, and fetal distress, twice as often as women without history of trauma (OR 2.13, p < 0.05). However, Blackmore et al. reported similar results as our study as their study also showed no association between trauma and obstetric complications [37].

The previous Finnish registry study also shows that the connections between different background factors to complications during pregnancy and childbirth are complex and would require more detailed research with sufficiently large data [9]. Furthermore, Väisänen et al. [9] study showed that socioeconomic factors could have some interactions with complications. It would have been useful to study such interactions also in this study, but the data of this study was not large enough.

In this study, 64% of women of Somali-origin and 32% of women of Kurdish-origin had undergone FGM/C. In both groups, the proportions were lower than the average in the women’s countries of origin. Although no associations between women with FGM/C and reproductive health complications were found in this study, previous studies have shown that FGM/C has severe immediate and long-term mental and physical health consequences in women’s life [31].

Guðmundsdóttir et al. [38] have presented that women’s citizenship and country of origin are significantly associated with a range of maternal and perinatal complications and interventions, such as episiotomy and instrumental birth. Especially women with a refugee background may not have equal access to care in their host countries. This might be related to many things: lack of health knowledge, unrecognized need of care by health professionals, fear and mistrust on authorities, experienced care-related discrimination, and care professionals’ negative attitudes and behavior [39]. In this study, access to services was good for Somali and Kurdish origin women and there was no difference in the number of visits between women who have experienced a potentially traumatic experiences and women with FGM/C compared to other women.

The average number of outpatient and inpatient visits among Somali- and Kurdish-origin women (13 among Somali and 16 among Kurdish origin women during their latest pregnancy) corresponds to the average number of visits (13–17 visits) among all pregnant women in Finland in recent years [40]. Kurdish-origin women had more outpatient and inpatient visits, probably because among Kurdish-origin women there are more women expecting their first child, in which case there are more visits. A functioning service system is likely to prevent avoidable complications during pregnancy and childbirth [41].

There were few women with diagnosed mental health disorders in this study. On the other hand, women with migrant origin may possibly have undiagnosed mental health problems. Previous studies indicate that women who belong to a minority ethnic group, have a higher risk for various mental health problems such as posttraumatic stress, anxiety, and prenatal psychosomatic symptoms [4], and prenatal as well as [42] post-partum depression [43]. A previous Finnish study showed that people with an immigrant background used less mental health services than people of Finnish-origin [44].

More research is needed on women with FGM/C because the psychological impact of FGM/C on women’s mental health is not well described. There is a need to develop effective, culturally appropriate treatment modalities for women living with or being surrounded by people who have undergone FGM/C [24].

Maternity health service staff need to be attuned to women’s psychological needs [45]. Risk groups should be identified at an early stage so that the appropriate treatment could be planned individually. Although the results of this and previous studies on the risks of reproductive health problems for women of a migrant origin vary, these women would benefit from more targeted care during pregnancy and childbirth, even in low-risk settings [46].

### Strengths and limitations

The strength of this study is the use of both survey and registry data. For this reason, the results are probably not influenced by the recall biases that might occur in surveys. Another strength of this study is that the register data are from several different sources, and therefore as comprehensive as possible. However, the information on the type of FGM/C could not be obtained in this study, and this is likely to differ between Somali [27]and Kurdish origin women [28]. If the type of FGM/C is less severe, this could explain the weak associations, especially for the Kurdish women. Survey data were linked to register data up to about 6–7 years after conducting the survey. The use of both survey and register data allows us to study of events that cannot be studied using only register data.

The limited number of women in this study restricted the analyses and affect the results, as shown in the wide confidence intervals. Selective non-response can cause some bias, especially in the Somali-origin group which had the lowest response rate [32]. Because of the small number of participants, we had to combine different complication diagnoses (item “any complications”) for the analyses. The results may also have been affected by various confounding factors (such as psychological, social, economic, demographic, health, and behavioral factors) that could not be ruled out in this study. Hypertension during pregnancy and gestational diabetes were included in the analyses at the beginning but were later excluded as there were no significant associations between these conditions and trauma experiences nor FGM/C. No significant differences were observed between Somali- and Kurdish-origin women.

One problem was that the exact timing of the exposures and the outcomes could not be determined accurately. Some of the self-reported miscarriages and induced abortions in the pregnancy history may have occurred before the actual potentially traumatic experience. There may also be a relatively long period between the time of the potentially traumatic experiences and the studied outcomes, mitigating the potentially traumatic experience. Despite these factors, it must also be considered that there was actually no association between potentially traumatic experiences and complications, which may indicate that potentially traumatic experiences have been adequately addressed in maternity care and other services.

The data was examined for several socioeconomic (SES) variables separately and the variable’s relationship to the outcome. In the interaction analysis, all combinations between the four dependent variables (miscarriages, induced abortions, complications during pregnancy, complications during birth), two explanatory variables (potentially traumatic experience and FGM/C) and three socio-demographic factors (marital status, education, work status) were examined. Our analysis gave weak indication of interactions, but due to very small cell numbers (zero or < 5 cases), it was impossible to draw any conclusions from the interaction analyses.

Since different studies give conflicting results about the connections between potentially traumatic experiences and complications, there is a need for further research. In addition, different studies use different methods to measure potentially traumatic experiences, and the other studies have also had small data sets with low statistical power, which can lead to studies giving different results.

## Conclusions

Potentially traumatic experiences and FGM/C were common in both groups, but they did not increase the risk of adverse reproductive health outcomes in this study. This study indicates that migrant Somali- and Kurdish-origin women may be vulnerable due to potentially traumatic experiences, and it is important to pay attention to the encounter and care of these women in reproductive health services.

### Electronic supplementary material

Below is the link to the electronic supplementary material.

Supplementary Material 1

## Data Availability

The data that support the findings of this study can be obtained from THL but restrictions apply due to data protection regulations, ethics approval of the surveys and the subjects` consent to participate. Thus the data are not publicly available. The data are however available following the guidelines at the survey website: https://thl.fi/en/web/thlfi-en/research-and-development/research-and-projects/migrant-health-and-wellbeing-study-maamu-/information%20for-researchers.
