# Supplementary material for: Potentially traumatic experiences pre-migration and adverse pregnancy and childbirth outcomes among women of Somali- and Kurdish-origin in Finland
Source: BMC Pregnancy Childbirth. 2023 Aug 17;23:589. doi: 10.1186/s12884-023-05906-w (PMC10433655; doi:10.1186/s12884-023-05906-w)
Supplement: Supplementary file 1 — Supplementary Material 1 [file 12884_2023_5906_MOESM1_ESM.docx]

**Appendix 1, Table 1. Detailed list of identified and included complications**

| **Variable name** | **^†^Diagnosis number and  ^¥^ *ICPC codes*** | **Classification** | **Data source** |
| --- | --- | --- | --- |
| **Exposure** | | | |
| Potentially traumatic experience pre-migration |  | trauma  no trauma | Self-reported (Maamu survey) |
| Female genital mutilation/cutting | Diagnoses related to female genitals that may indicate female genital mutilation:  Z41.2, N90.8, O34.7, N99.9, N99.8, S38.2, N99.1, N99.2, S30.2, S31.4, S31.5, O34.6, O34.7, O34.8, O34.8,  *W81, W84, W85, T89, T90, K85, K86, K87* | yes FGM/C  no FGM/C | Combined data: Self-reported (Maamu survey) and *register data |
| **Confounding factors** | | | |
| Age when moved to Finland | - | ≤5 yrs  6–14 yrs  ≥15 yrs | Combined data: Self-reported and *register data |
| Age of the mother at the time of the last reproductive health event  (pregnancy /birth) | - | - | Combined data:  Self-reported (Maamu survey) and *register data |
| Body mass index (BMI) ≥25 | - | continuous and binary variable (yes / no) | Health examination data,  measured (Maamu survey) |
| Hypertension during pregnancy | -Edema in pregnancy and proteinuria, no hypertension, -Hypertension in pregnancy, no proteinuria and -Hypertension in pregnancy and proteinuria (pre-eclampsia),  -Eclampsia  O12.0–O12.2, O13, O14.0–O14.9, O15.0–O15.2, O15.9, O16  *W81* | yes hypertension during pregnancy  no hypertension during pregnancy | *Register data:  Medical Birth Register,  The Care Register for Health Care The Primary Health Care Register |
| Gestational Diabetes | Diabetes mellitus in pregnancy (GDM)  O24.4, O24.9  W84, W 85 |  | *Register data:  Medical Birth Register,  The Care Register for Health Care, The Primary Health Care Register |
| Number of births | Delivery  O80-O84 *W90* | no births  1–2 birth  ≥3 births | Combined self-reported and *register data:  Medical Birth Register, The Care Register for Health Care, The Primary Health Care Register |
| **Outcomes** | | | |
| Miscarriages | Pregnancy with abortive outcome O00–O03, O06 & O08, N96  *W80, W82* | none  1–2  ≥3  and  at least one | Combined data:  Self- reported (Maamu survey)  and / or *registered in Finland |
| Induced abortions | Medical abortion O04-O05, O07  *W83* | none  one  ≥ 2  and  at least one | Combined data:  Self- reported  and / or *registered in Finland |
| Complications  during pregnancy at first trimester | Bleeding (at first trimester)  O20, O46  W03 | yes complications no complications | *Register data:  Medical Birth Register, The Care Register for Health Care, The Primary Health Care Register |
| Complications during pregnancy related to fetus | -Known or suspected fetal abnormality and damage  O35.0–35.9  W84  -Signs of fetal hypoxia  O36.3  W84  -Death of fetus  O36.4  W93  -Other fetal problems  O36.5–O36.9  W99 | yes complications no complications | *Register data:  Medical Birth Register, The Care Register for Health Care, The Primary Health Care Register |
| Other complications during pregnancy | -Premature rupture of membrane (before 37 weeks)  O42.0–O42.9  W92  -Bleeding before birth  O46.8–O46.9  W03  -False labour (contractions)  O47.0–O47.9  W99 | yes complications no complications | *Register data:  Medical Birth Register, The Care Register for Health Care, The Primary Health Care Register |
| Complications during pregnancy and birth | -Preterm birth  O60  W92  -Failed induction of labor  O61.0–O63.9  W92  -Labor and delivery complicated by intrapartum hemorrhage  O67.0–O67.9  W92  -Fear of childbirth  O99.80  W84  follow up, risk pregnancy  Z35.0–Z35.9  W84 | yes complications no complications | *Register data:  Medical Birth Register, The Care Register for Health Care, The Primary Health Care Register |
| Complications during birth, tears, haemorrage and other | -Perineal tear during childbirth O70.2–O71.9 *W92* -Haemorrhagia post-partum O72.0–O72.3 *W17* -Complications of labor and delivery O75.0–O75.7 *W71* -Other specified complications of labor and delivery O75.8–O75.9 *W92* | yes complications no complications | *Register data:  Medical Birth Register, The Care Register for Health Care, The Primary Health Care Register |
| Complications during birth, assisted birth and other | -Assisted birth  O81.0–O83.9  W92  -Sequelae of complications of pregnancy, childbirth, and the puerperium  O94.0–O97.0  W99  -Follow up, risk pregnancy  Z35.0–Z35.9  W84  -Stillbirth  Z37.1, Z37.9  W91 | yes complications no complications | *Register data:  Medical Birth Register, The Care Register for Health Care, The Primary Health Care Register |
| Complications at any pregnancy event | *pregnancy complication diagnosis, any of the above* | yes complications no complications | *Register data:  Medical Birth Register, The Care Register for Health Care, The Primary Health Care Register |
| Complications at any birth event | *birth complication diagnosis,* *any of the above* | yes complications no complications | *Register data:  Medical Birth Register, The Care Register for Health Care, The Primary Health Care Register |

† International classification of diseases

¥ International classification of primary care

*The Register for Outpatient Visits in Primary Care (AvoHilmo) (diagnosis from year 2011–2018) The Care Register for Health Care (Hilmo) (diagnosis from year 1994–2018) + The Finnish Medical Birth Register (diagnosis from year 1987–2018) + Register of Induced Abortions (diagnosis from years 1983–2018)
